# Exposure to Nerve Growth Factor Worsens Nephrotoxic Effect Induced by Cyclosporine A in HK-2 Cells

**DOI:** 10.1371/journal.pone.0080113

**Published:** 2013-11-07

**Authors:** Donatella Vizza, Anna Perri, Danilo Lofaro, Giuseppina Toteda, Simona Lupinacci, Francesca Leone, Paolo Gigliotti, Teresa Papalia, Renzo Bonofiglio

**Affiliations:** Kidney and Transplantation Research Center, Department of Nephrology, Dialysis and Transplantation, “Annunziata” Hospital, Cosenza, Italy; University of Houston, United States of America

## Abstract

Nerve growth factor is a neurotrophin that promotes cell growth, differentiation, survival and death through two different receptors: TrkA^NTR^ and p75^NTR^. Nerve growth factor serum concentrations increase during many inflammatory and autoimmune diseases, glomerulonephritis, chronic kidney disease, end-stage renal disease and, particularly, in renal transplant. Considering that nerve growth factor exerts beneficial effects in the treatment of major central and peripheral neurodegenerative diseases, skin and corneal ulcers, we asked whether nerve growth factor could also exert a role in Cyclosporine A-induced graft nephrotoxicity. Our hypothesis was raised from basic evidence indicating that Cyclosporine A-inhibition of calcineurin-NFAT pathway increases nerve growth factor expression levels. Therefore, we investigated the involvement of nerve growth factor and its receptors in the damage exerted by Cyclosporine A in tubular renal cells, HK-2. Our results showed that in HK-2 cells combined treatment with Cyclosporine A + nerve growth factor induced a significant reduction in cell vitality concomitant with a down-regulation of Cyclin D1 and up-regulation of p21 levels respect to cells treated with Cyclosporine A alone. Moreover functional experiments showed that the co-treatment significantly up-regulated human p21promoter activity by involvement of the Sp1 transcription factor, whose nuclear content was negatively regulated by activated NFATc1. In addition we observed that the combined exposure to Cyclosporine A + nerve growth factor promoted an up-regulation of p75 ^NTR^ and its target genes, p53 and BAD leading to the activation of intrinsic apoptosis. Finally, the chemical inhibition of p75^NTR^ down-regulated the intrinsic apoptotic signal. We describe two new mechanisms by which nerve growth factor promotes growth arrest and apoptosis in tubular renal cells exposed to Cyclosporine A.

## Introduction

Nerve growth factor (NGF) is a neurotrophin produced and released by a number of different mammalian cells acting on cell survival and differentiation, tissue repair and inflammatory responses [1]. NGF exerts its biological effects through binding to two distinct classes of cell surface receptors: the specific NGF neurotrophic tyrosine kinase receptor type 1 (TrkA^NTR^) and the pan-neurotrophin low affinity glycoprotein receptor (p75^NTR^), a typical death receptor belonging to the tumor necrosis receptor superfamily [2]. A specific cell-surface TrkA ^NTR^ / p75^NTR^ ratio seems to be directly responsible for either proliferative and/or survival effects (TrkA ^NTR^) or apoptotic responses (p75 ^NTR^), with p75^NTR^ acting alone or in combination to modulate TrkA ^NTR^ trafficking and/or signaling [2]. NGF serum concentrations change during inflammation and inflammatory mediators induce NGF synthesis in a variety of cell types, although why NGF concentration is enhanced and how this can affect inflammatory responses are far from being fully understood [1]. We previously reported elevated serum NGF levels in patients affected by glomerulonephritis, chronic kidney disease and end-stage renal disease even though we did not explore the significance of our findings [3]. Recently, in a cohort of renal transplant recipients we found higher NGF serum levels respect to healthy controls [4]. Interestingly, the observed NGF levels were higher than those detected in other kidney diseases investigated [3,4].

Cyclosporine A (CsA) is an immunosuppressive drug belonging to the calcineurin inhibitor (CNIs) family commonly used to prevent acute rejection in solid organ transplantation [5,6]. Its immunosuppressive action is mediated through preventing T-cell activation inhibiting the transcriptional activation of interleukin 2 and 4 genes [7]. However, CNIs have side effects such as inducing nephrotoxicity [8], hypertension [9] and dyslipidemia [10], contributing to Chronic Allograft Dysfunction pathogenesis, through molecular mechanisms not yet completely understood [11-13]. Therefore, some *in vivo* and *in vitro* studies have demonstrated the advantages and disadvantages of using additional drugs to counteract the CsA side effect [14-16].

The best-described substrates of CNIs are NFAT (Nuclear Factor of Activated T Cells) family transcription factors. At present five NFAT isoforms are known (NFAT types c1 to c4 and NFAT5) which, by their nuclear translocation, regulate the expression of different genes, including signaling proteins, cytokines, cell surface receptors and cell cycle or apoptosis related proteins [17,18]. Recently Rana et al, using an *in vitro* model of rat cardiomyocytes, demonstrated that the calcineurin-NFAT pathway decreased NGF protein and gene expression and that treatment with CNIs, via NFAT-inhibition, resulted in a significant increase of NGF protein levels by a feedback mechanism [19]. Considering that NGF acts as modulator of cell survival, tissue repair and inflammatory response and that it is also modulated by the calcineurin NFAT-pathway, it is reasonable that NGF could also exert a role in graft nephrotoxicity induced by CNIs. In this context, the aims of the present study are (i) to verify, using an *in vitro* model of proximal tubular renal cells (HK-2), whether exposure to CsA modulates NGF expression; (ii) investigate in the same experimental conditions the role of NGF in CNI-induced tubular cell damage.

## Materials and Methods

### Cell culture

Human renal proximal tubular cells, HK-2, immortalized with HPV-16 were cultured in Keratinocyte-SFM supplemented with 5 ng/ml Epidermal Growth Factor (EGF), 0.05 mg/ml bovine pituitary extract (BPE) and 1 mg/ml penicillin/streptomycin (P/S). Before each experiment, cells were starved in serum-free medium containing 5% of complete medium for 24 h and then treated as described.

### Chemicals

NGF was obtained from Invitrogen (Milano) and solubilized in DNA/RNAase free water.

Cyclosporine A (CsA), Phorbol 12-myristate 13-acetate (PMA), calcium ionophore A23187 (Io) and Mithramycin (M) were purchased from Sigma Aldrich (Milan, Italy) and solubilized in pure ethanol. 

### Western Blot Analysis

Cells were grown in 6 cm dishes to 70–80% confluence and exposed to treatments in serum free medium supplemented with 5% of complete medium as indicated. Cells were harvested in cold phosphate-buffered saline (PBS) and resuspended in total RIPA buffer containing 1% NP40, 0.5% Na-deoxycholate, 0.1% SDS and inhibitors (0.1mM sodium orthovanadate, 1% phenylmethylsulfonylfluoride or PMSF, 20mg/ml aprotinin). Protein concentration was determined by Bio-Rad Protein Assay (Bio-Rad Laboratories, Hercules, CA USA). A total of 40 μg of total lysates was used for western blotting (WB), resolved on 8% or 11% SDS-polyacrylamide gel, transferred to a nitrocellulose membrane and probed with antibodies directed against the TrkA ^NTR^ (1:300), p75^NTR^ (1:300), NGF (1:300), Cyclin D1 (CD1) (1:500), p21 (1:500) (Santa Cruz Biotechnology, CA USA), the phosphorylated and total forms of MAPK (1:1000), AKT (1:1000), mTOR (1:500) and JNK (1:200) (Cell Signaling Technology, Milan, Italy). As internal control, all membranes were subsequently stripped (0.2 M glycine, pH 2.6, for 30 min at room temperature) of the first antibody and reprobed with anti-β-actin antibody (1:10000) (Santa Cruz Biotechnology). The antigen-antibody complex was detected by incubation of the membranes for 1 h at room temperature with peroxidase-coupled goat anti-mouse (1:2000) or anti-rabbit (1:7000) or donkey anti-goat (1:3000) IgG and revealed using the enhanced chemiluminescence system (Amersham Pharmacia, Buckinghamshire UK). Blots were then exposed to film (Kodak film, Sigma).

To obtain cytosolic nuclear fraction of proteins, cells were grown in 10 cm dishes to 70–80% confluence and, after synchronization for 24 h, exposed to treatments as indicated. Cells were washed in cold PBS and then 300μl of cytosolic buffer plus 10μg/ml aprotinin, 50mM PMSF and 50mM sodium orthovanadate were added in each plate. Cells were incubated for 5 min at 4°C and centrifuged at 13,000 rpm for 10min at 4°C.The supernatant, containing the cytosolic protein fraction, was collected, while the resulting cells were collected, resuspended in a nuclear buffer containing 20mM HEPES pH 8, 0.1mM EDTA, 5mM MgCl_2_, 0.5M NaCl, 20% glycerol, 1% NP-40, inhibitors and then incubated over night at 4°C. The next day extracts were centrifuged at 12,000 rpm for 10 min at 4°C and supernatants were recovered. Equal amounts of cytosolic and nuclear proteins (60 μg) were resolved by 8% SDS-PAGE and probed with antibodies directed against NFATc1 (Atgen) (1:100), Sp1 (1: 1000), β-actin (1:10000) and Lamin B (1: 10000) (Santa Cruz Biotechnology). 

### RT–PCR and Real-time RT–PCR assays

HK-2 cells were grown in 10 cm dishes to 70–80% confluence, and exposed to treatments as indicated. Total cellular RNA was extracted using TRIZOL reagent (Invitrogen) as suggested by the manufacturer. The purity and integrity of the RNA was confirmed both spectroscopically and electrophoretically. RNA was then reversed transcribed with High Capacity cDNA Reverse Transcription Kit (Applied Biosystems, Applera Italia, Monza, Milano, Italy). Analysis of CD1, p21, NFATc1, NFATc2, NFATc3, NFATc4, NFATc5, p75 ^NTR^, TrkA ^NTR^, p53, BAD and Bcl-2 gene expression was performed using Real-time reverse transcription PCR. cDNA was diluted 1:3 in nuclease-free water and 5μl were analyzed in triplicates by real-time PCR in an iCycler iQ Detection System (Bio-Rad, USA) using SYBR Green Universal PCR Master Mix with 0.1mmol/l of each primer in a total volume of 30μl reaction mixture following the manufacturer’s recommendations. Each sample was normalized on its GAPDH mRNA content. Relative gene expression levels were normalized to the basal, untreated sample chosen as calibrator.

Final results are expressed as fold difference in gene expression relative to GAPDH mRNA and calibrator, calculated following the ΔCt method, as follows: Relative expression (folds) = 2-(ΔCtsample-ΔCtcalibrator) where ΔCt values of the sample and calibrator were determined by subtracting the average Ct value of the GAPDH mRNA reference gene from the average Ct value of the analyzed gene. For CD1, p21, TrkA ^NTR^, p75 ^NTR^, p53, BAD, Bcl-2, NFATc1, NFATc2, NFATc3, NFATc4 and NFAT5 the primers used for the amplification were:

Cyclin D1: forward 5’-GATGCCAACCTCCTCAACGAC-3’ and reverse 5’-CTCCTCGCACTTCTGTTCCTC-3’
p21: forward 5’-GCAGACCAGCATGACAGATTT-3’ and reverse 5’-GGATTAGGGCTTCCTCTTGGA-3’
TrkA ^NTR^: forward 5’- CATCGTGAAGAGTGGTCTCCG-3’ and reverse 5’-GAGAGAGACTCCAGAGCGTTGAA-3’
p75^NTR^: forward 5’-CCTACGGCTACTACCAGGATGAG-3’ and reverse 5’-TGGCCTCGTCGGAATACG-3’
p53: forward 5’-GCTGCTCAGATAGCGATGGTC-3’ and reverse 5’-CTCCCAGGACAGGCACAAACA-3’
BAD: forward 5’-AGCCAACCAGCAGCAGCCATCAT and reverse 5’-CTCCCCCATCCCTTCGTCGTC-3’;
Bcl-2: forward 5’-GGGGAGGATTGTGGCCTTC-3’ and reverse 5’-CAGGGCGATGTTGTCCACC-3’
NFATc1: forward 5’- CTGTGCAAGCCGAATTCTCTGG-3’ and reverse 5’- ACTGACGTGAACGGGGCTGG-3’
NFATc2: forward 5’- AAGAGCCAGCCCAACATGC -3’ and reverse 5’- CGTTTTCTCTTCCCATTGATGAC -3’
NFATc3: forward 5’- GCGGCCTGCAGATCTTGAGC -3’ and reverse 5’- TGATGTGGTAAGCAAAGTGGTGTGGT -3’
NFATc4: forward 5’- GTCCTGATGGGAAGCTGCAATGG -3’ and reverse 5’- AGCGTCACCTCGTTGCTCTGC -3’
NFAT5: forward 5’- GACACTGGCGGTGGACTGCG -3’ and reverse 5’- CTGGCTTCGACATCAGCATTCCT -3’

Negative controls contained water instead of first strand cDNA.

### RNA interference (RNAi)

Cells were plated in 6 cm dishes with regular growth medium the day before transfection to 60–70% confluence. On the second day the medium was changed with serum free medium plus 5% of complete medium without P/S and cells were transfected with stealth RNAi targeted human p75^NTR^ mRNA sequence 5'-UGG ACA GCC AGA GCC UGC AUGACCA-3' (Invitrogen), or with a stealth RNAi-negative control (Invitrogen) to a final concentration of 100nM using Lipofectamine 2000 (Invitrogen) as recommended by the manufacturer. After 5h the transfection medium was changed with the same medium mentioned above supplemented with P/S in order to avoid Lipofectamine 2000 toxicity, cells were exposed to treatments and subjected to different experiments.

### MTT assay

Cell viability was determined with the 3-(4,5-dimethylthiazol-2-yl)-2,5 diphenyltetrazolium (MTT) assay. HK-2 cells (4X10^6^cells/ml) were grown in 24 well plates and exposed to treatments as indicated in serum free medium added with 5% of complete medium. 100µl of MTT (2mg/ml, Sigma) were added to each well, and the plates were incubated for 2 h at 37°C. Then, pure DMSO was added to solubilize cells. The absorbance was measured at a test wavelength of 570 nm in Beckman Coulter microplate reader.

### Transient transfection assay

HK-2 cells were transfected in Serum Free Medium (SFM) supplemented with 5% of complete medium using the FuGENE 6 reagent as recommended by the manufacturer (Roche Diagnostics, Mannheim, Germany) with a mixture containing 0.5 µg of the human wild-type p21^Cip1/WAF1^ promoter-luciferase (luc) reporter (p21Cip1/WAF1 wt) and 5 ng of pRL-CMV (Promega), which expresses Renilla luciferase enzymatically distinguishable from firefly luciferase by the strong cytomegalovirus enhancer/promoter. 24 h after transfection, the cells were untreated or treated with CsA, NGF and Mithramycin alone or in combination for 15 h. Firefly and Renilla luciferase activities were measured using the Dual Luciferase kit. The firefly luciferase values for each sample were normalized based on the transfection efficiency measured by Renilla luciferase activity. Data were reported as fold induction respect to control.

### Immunoprecipitation

300μg of nuclear and cytosolic total extracts from HK-2 cells proteins were incubated overnight with 2μg of anti-Sp1 antibody and 500μL of HNTG (immunoprecipitation buffer [50 mmol/L HEPES (pH 7.4), 50 mmol/L NaCl, 0.1% Triton X-100, 10% glycerol, 1mmol/L phenylmethylsulfonylfluoride, 10μg/mL leupeptin, 10μg/mL aprotinin, 2μg/mL pepstatin]). Immunocomplexes were recovered by incubation with protein A/G-agarose. The immunoprecipitates were washed with HNTG buffer and subjected to SDS–polyacrylamide gel electrophoresis. Equal amounts of cell extracts were subjected to SDS–polyacrylamide gel electrophoresis. Membranes were probed with anti-NFATC1 and anti Sp1antibodies. The bands of interest were quantified by Image J densitometry scanning program.

### Caspase activity

HK-2 cells were plated in complete culture medium, starved for 24h in serum free medium supplemented with 5% of complete medium without P/S and then transfected with p75 RNAi or with a stealth RNAi-negative control (Invitrogen) to a final concentration of 100nM using Lipofectamine 2000 (Invitrogen) as recommended by the manufacturer. After 5h the transfection medium was changed with the same medium mentioned above supplemented with P/S in order to avoid Lipofectamine 2000 toxicity, cells were exposed to treatments for 72h. Cells were trypsinized, 5X10^6^ cells were counted, resuspended in 50µl of chilled Cell Lysis Buffer and incubated on ice for 10 min. Each sample was centrifuged for 1 min (10.000 rpm). Supernatants were transferred to a fresh tube and protein concentration was measured. 200µg of proteins were used. Next, a mixture containing 50µl of 2X Reaction Buffer plus 10mM DTT and 5µl of LEGD-Pna substrate was added to each sample. Tubes were incubated at 37 °C in the dark for 2 h. Caspase activity was measured at 400nm in a microplate reader and was reported as fold induction respect to untreated cells.

### Statistical analysis

All experiments were performed in at least triplicate per treatment and repeated in three independent experiments. MTT and Real Time RT-PCR results are presented as fold induction over basal condition. Optical densities were measured using the Scion Image software (Scion Corporation) and their results are presented as percentage respect to control. All results are presented as mean ± SD of data from three combined experiments. Data were analyzed by unpaired t-test (between two groups) or one-way analysis of variance with Tukey or Dunnett post-test analysis (for three or more groups) using R (Version 2.15.1, R Core Team); a p value < 0.05 was considered significant. 

## Results

### CsA treatment enhances NGF expression via NFATc1 in HK-2 cells

**Figure 1:**
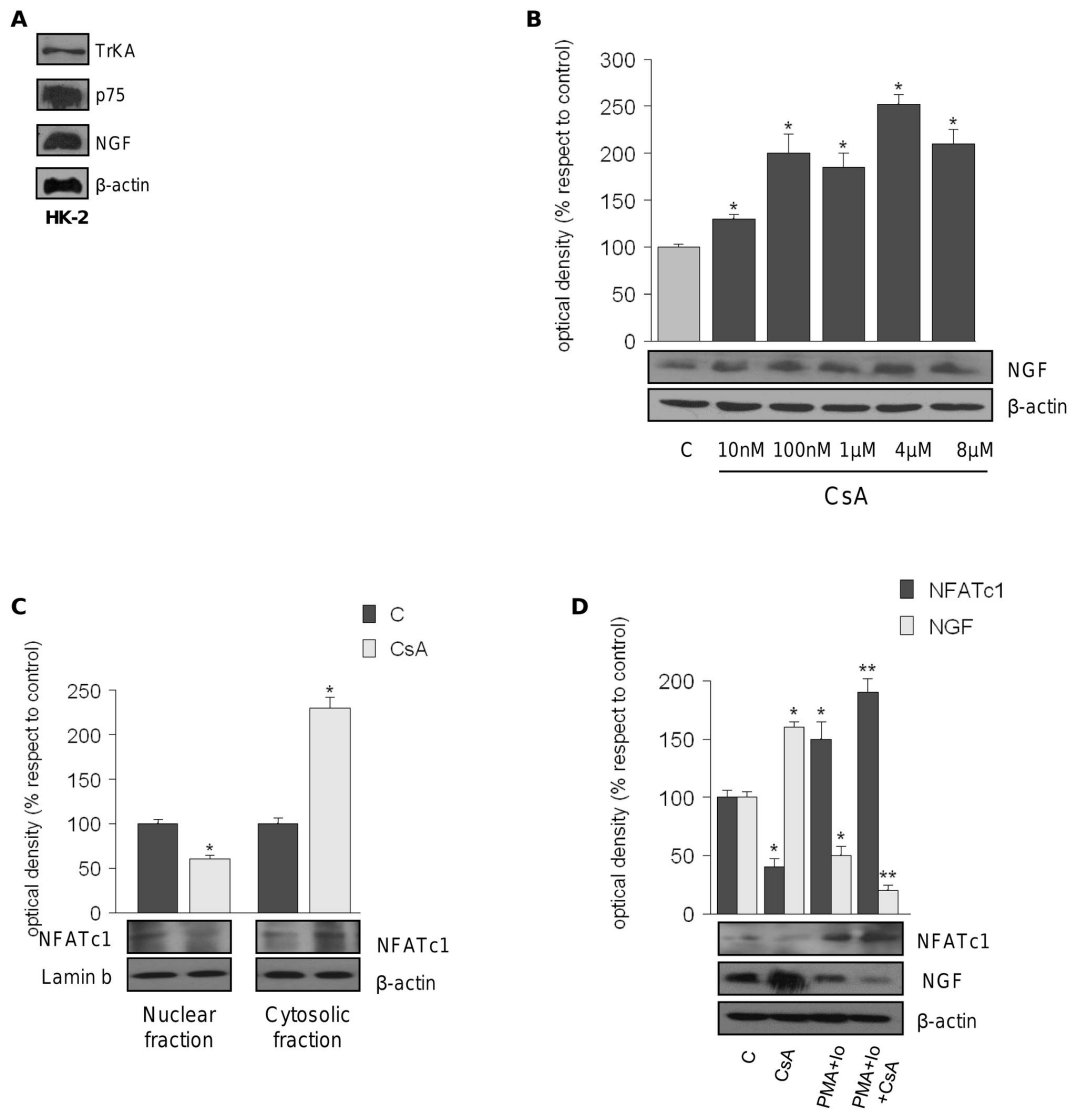
CSA up-regulates NGF protein levels via NFATc1. (A) WB of TrKA^NTR^ (Antibody dilution 1:300), p75^NTR^ (1:300) and NGF (1:300) basal protein expression in HK-2 cells. β-Actin (1:10000) was used as loading control. (B) HK-2 cells were untreated (c) or treated for 48h with CsA 10nM-100nM-1μM-4μM-8μM before lysis. Equal amounts of total cellular extract were analyzed for NGF levels by western blotting. β-Actin was used as loading control. Bars represent the means ± SD of 3 separate experiments between NGF/β-Actin levels in which band intensities were evaluated as density arbitrary units and expressed as percentages of the control (100%). *p<0.05 vs c. (C) HK-2 cells were treated with CsA 10nM for 3h. Nuclear and cytosolic fractions were analyzed by immunoblotting using anti-NFATc1 Ab (1:100). β-Actin and Lamin B (1:10000) were used respectively as cytosolic and nuclear loading controls. Immunoblots show a single representative of 3 separate experiments. The upper panels represent the means ± SD of 3 separate experiments between NFATc1/β-Actin and NFATc1/Lamin B levels in which band intensities were evaluated as optical density arbitrary units and expressed as percentages of the control which was assumed to be 100%. *p<0.05 vs c. (D) WB of NFATc1 and NGF in total extracts from HK-2 cells treated with CsA 10nM, PMA 100nM+Io 2.5μM and CsA+PMA+Io β-Actin was used as loading control. The upper panels represent the means ± SD of 3 separate experiments between NFATc1/β-Actin and NGF/β-Actin levels in which band intensities were evaluated in terms of optical density arbitrary units and expressed as percentages of the control (100%). *p<0.05 vs c; **p<0.05 vs CsA-treated cells. All immunoblots show a single representative of 3 separate experiments. Secondary antibodies dilutions used are: goat anti-mouse (1:2000) or anti-rabbit (1:7000) or donkey anti-goat (1:3000) IgG.

In the first step of our *in vitro* studies we demonstrated that HK-2 cells constitutively express NGF as well as its receptors (Figure 1A). Next, to evaluate whether CsA modulates NGF levels, we treated HK-2 cells with CsA for 48 h. Our results showed that the protein expression levels of NGF begin to increase upon 10nM CsA treatment (+ 30 ± 3.45%) and that the up-regulation was maintained up to 8µM (+110 ± 15.0%) (Figure 1 B). 

To explore the role of the calcineurin-NFAT pathway in the NGF up-regulation observed upon CsA treatment, we first examined the expression levels of the five NFAT isoforms in our cellular system. RT-PCR assay revealed that NFATc1 is the main isoform expressed in HK-2 cells (data not shown). As expected, using nuclear and cytosolic extracts obtained from HK-2 cells exposed to short treatment with CsA, we observed that CNI increased cytosolic inactivated NFATc1 content up to 220 ± 12.0 %, reducing the nuclear activated fraction (-33.2 ± 3.42 %,Figure 1C). 

According to that reported in literature [12],to verify that in our cellular system CsA increases the NGF levels via NFATc1 inhibition, HK-2 cells treated with CsA for 48 h, were pre-treated with phorbol 12-myristate 13-acetate (PMA) plus calcium ionophore A23187 (Io) to induce NFATc1 activation. As expected, the exposure to PMA plus Io, increased NFATc1 levels of 50 ± 15.0 %; interestingly, in HK-2 cells treated with NFATc1 activators, the exposure to CsA did not increased NGF protein levels, indeed we observed that upon PMA+Io+CsA NGF expression was significantly decreased (-80 ± 4.50 %), emphasizing that CsA increases NGF levels via NFATc1 (Figure 1D). 

### The combined treatment CsA plus NGF increases p21 expression and its promoter activity via Sp1, inducing HK-2 cell growth arrest

**Figure 2:**
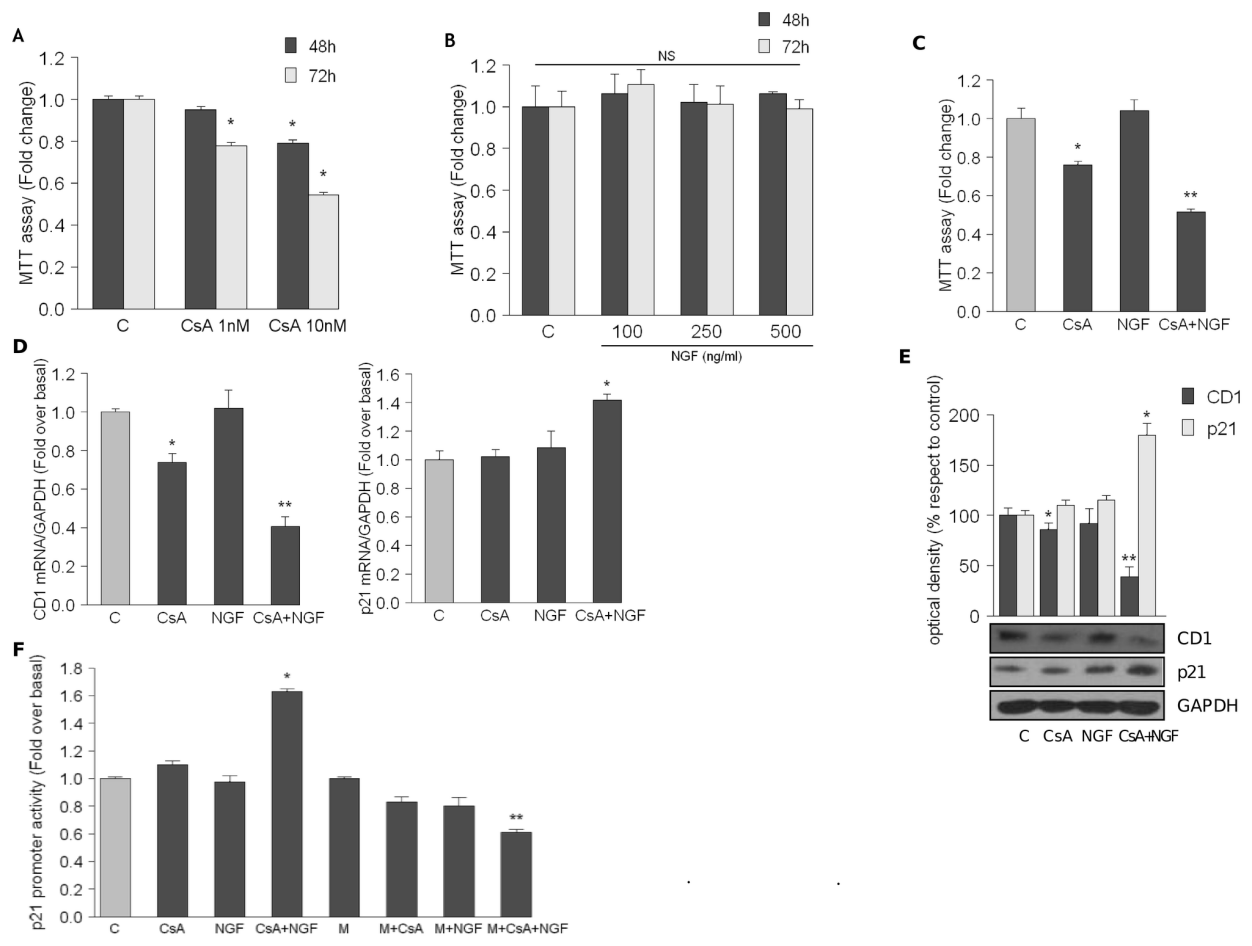
Combined treatment with CsA plus NGF inhibits cell growth inducing p21 promoter activity. (A) MTT growth assays in HK-2 cells untreated (c) or treated with CsA (1–10nM) (A) or NGF (100–250–500ng/ml) (B). Cell proliferation is expressed as fold change respect to control. *p<0.05 vs c ; NS: not significant. (C) MTT growth assay in HK-2 cells untreated (c) or treated with CsA 10nM, NGF 100 ng/ml, alone or in combination for 48h. Results are expressed as fold change respect to c. *p<0.05 compared with c. **p<0.05 compared with CsA-treated cells. (D) CD1 and p21 mRNA content, evaluated by real time RT-PCR after 24 hours of exposure to CsA 10nM and NGF 100ng/ml alone or in combination. Each sample was normalized to its GAPDH mRNA content. *p<0.05 compared with c; **p<0.05 compared with CsA-treated cells. (E) HK-2 cells were untreated (c) or treated for 24h with CsA 10nM and NGF 100ng/ml alone or in combination. CD1 (Antibody dilution 1:500) and p21 (1:500) protein levels were determined by WB. β-Actin (1:10000) was used as loading control. Immunoblots show a single representative of 3 separate experiments. The upper panels represent the means ± SD of 3 separate experiments between CD1/β-Actin and p21/β-Actin levels in which band intensities were evaluated in terms of optical density arbitrary units and expressed as percentages of the control (100%). *p<0.05; **p<0.05 compared with CsA-treated cells. (F) HK-2 cells, transiently transfected with p21 promoter, were untreated (c) or treated for 15h with CsA 10nM, NGF 100ng/ml, Mithramycin (M) 100nM alone or in combination and then luciferase activity was measured. Luciferase activity of untreated cells was set as 1-fold induction, upon which treatments were calculated. *p<0.05 compared with c; **p<0.05 compared with cells CsA+NGF-treated. Bars represent the means ± SD of 3 different experiments each performed in triplicate. Secondary antibodies dilutions used are: goat anti-mouse (1:2000) or anti-rabbit (1:7000).

Prior to exploring the role of NGF in the cell damage observed in HK-2 cells exposed to CsA, we confirmed that CsA was able to reduce cell viability in a time and dose dependent-manner (Figure 2A). Moreover, to investigate whether NGF affects tubular renal cell growth, we treated cells with increasing doses of NGF. MTT assay revealed that NGF did not modify HK-2 cell viability (Figure 2B). However, subjecting HK-2 cells to combined exposure to CsA plus NGF for 48 h, we observed a further worsening in viable cell (0.5 ± 0.012, Figure 2C). Since cyclin D1 specifically associates with selected cyclin-dependent kinases phosphorylating the retinoblastoma protein 1 (Rb1) to modulate cell growth [20] we assessed its expression together with that of the main cyclin-dependent kinase inhibitor p21. HK-2 cells were incubated with CsA and NGF alone or in combination for 24 h prior to evaluation of cyclin D1 and p21 mRNA expression both by real-time RT-PCR and western blot analysis We observed that CsA treatment caused a decrease of mRNA (+0.74 ± 0.046) and protein expression (-15.0 ± 5.1%) of cyclin D1, without a significant modulation of p21 mRNA and protein expression (Figure 2D, 2E). Interestingly, the co-treatment CsA plus NGF produced a marked down-regulation of mRNA and protein cyclin D1 expression (0.40 ± 0.05 and -70% ± 3.5%) together with and up-regulation of mRNA and protein p21 expression (respectively: 1.40 ± 0.044 and +75% ± 5.0%) (Figure 2D, 2E). As it has been reported that both CsA and NGF induce the transcriptional activation of p21 [15-17], we tested in our cellular system whether the combined treatment, CsA plus NGF, might modulate p21 promoter activity, containing multiple responsive elements for different transcription factors, including Sp1. To this aim, HK-2 cells were transiently transfected for 24 h with a reporter plasmid containing the wild type human p21 promoter region and then treated for 15 h with CsA and NGF alone or in combination. Our results revealed that the co-treatment CsA plus NGF increased p21 promoter transcriptional activity up to 1.76 ± 0.02 (Figure 2F). Moreover, our findings revealed the involvement of the transcription factor Sp1 in the p21 promoter transactivation, since we observed a down-regulation of promoter transcriptional activity in cells pre-treated with mithramycin (0.62 ± 0.02), a selective inhibitor of Sp1 binding to its responsive elements within the p21 gene promoter (Figure 2F). 

**Figure 3:**
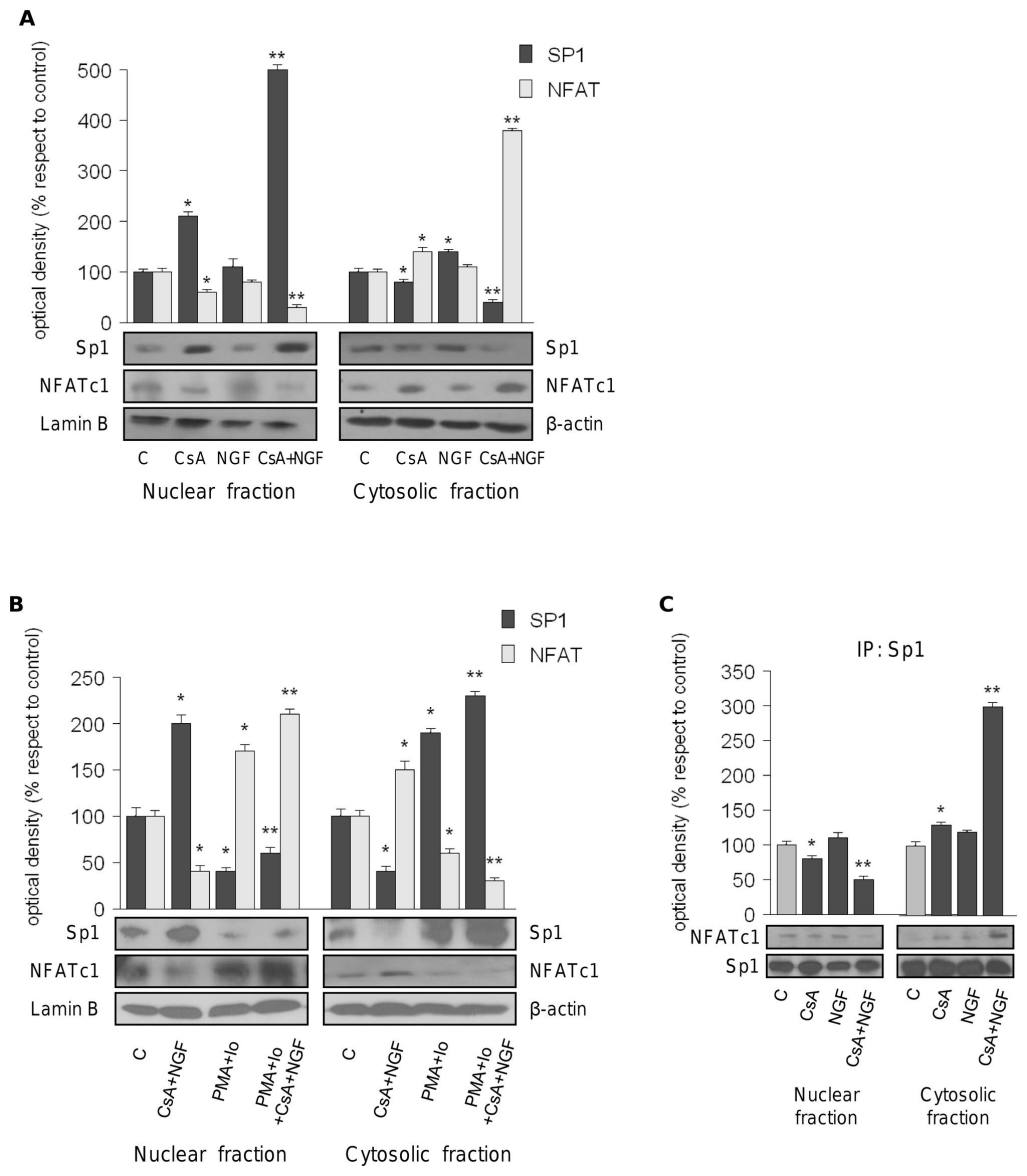
Activated NFATc1 negatively modulates Sp1 nuclear content. (A) HK-2 cells were untreated (c) or treated with CsA 10nM, NGF 100ng/ml alone or in combination for 15h before lysis. Nuclear and cytosolic expression of NFATc1 (Antibody dilution 1:100) and Sp1 (1:1000) were evaluated. Lamin B (1:10000) and β-Actin (1:10000) were used respectively as nuclear and cytosolic loading controls. The upper panels represent the means ± SD of 3 separate experiments between Sp1/Lamin B or Sp1/β-Actin and NFATc1/Lamin B or NFATc1/β-Actin levels in which band intensities were evaluated as optical density arbitrary units and expressed as percentages of the control (100%). *p<0.05 compared with c; **p<0.05 compared with cells treated with CsA alone (B) NFATc1 and Sp1 nuclear and cytosolic protein content of HK-2 cells untreated (c) or treated for 15 h with CsA 10nM+NGF 100ng/ml, PMA 100nM+Io 2.5μM, PMA+Io+CsA+NGF. The upper panels represent the means ± SD of 3 separate experiments between Sp1/Lamin B or Sp1/β-Actin and NFATc1/Lamin B or NFATc1/β-Actin levels in which band intensities were evaluated as optical density arbitrary units and expressed as percentages of the control (100%). *p<0.05 vs untreated cells. (C) HK-2 cells were untreated (c) or treated with CsA 10nM, NGF 100ng/ml alone or in combination for 24h. Total cell lysates were immunoprecipitated with anti-Sp1 antibody (IP:Sp1) and then subjected to immunoblot analyses with anti-NFATc1 and anti-Sp1 antibodies. The upper panels represent the means ± SD of 3 separate experiments between NFATc1/Sp1 levels in which band intensities were evaluated as optical density arbitrary units and expressed as percentages of the control (100%). *p<0.05 vs untreated cells; **p<0.05 vs cells CsA+NGF-treated. All immunoblots show a single representative of 3 separate experiments. Secondary antibodies dilutions used are: goat anti-mouse (1:2000) or anti-rabbit (1:7000) or donkey anti-goat (1:3000) IgG.

Previously it has been demonstrated that in human intestinal cells the activated NFATc1 acts as a negative regulator of Sp1 binding to the TRAIL (tumor necrosis factor-related apoptosis-inducing ligand) promoter [21]. To explore whether in our cellular model NFATc1 regulates Sp1 nuclear translocation and, consequently, the p21 promoter transactivation, we first analyzed the cellular localization of Sp1 and NFATc1 proteins extracted from cells treated with CsA and NGF alone or in combination for 15 h. As showed in Figure 3A, co-treatment of CsA plus NGF decreased nuclear NFATc1 protein levels of 70 ± 5.2% and increased the nuclear Sp1 fraction (+400 ± 4.59%), suggesting the negative regulation by activated NFATc1 on binding of Sp1 on p21 gene promoter. Moreover, to confirm the crucial role exerted by NFTAc1 in the Sp1 nuclear translocation, we determined Sp1 and NFTAc1 levels in nuclear and cytosolic compartment after treatment with PMA plus Io. Western blot analysis revealed that NFTAc1 activation by PMA and Io reduced Sp1 nuclear content (-60 ± 4.56%) and that the observed down-regulation persisted upon CsA plus NGF treatment (-40 ± 4.59%, Figure 3B). To better define the role of NFATc1 in the inhibition of Sp1 binding on p21 promoter we performed co-immunoprecipitation studies using cytosolic and nuclear extracts from HK-2 cells treated for 15 h with CsA and NGF alone or in combination. Equal amounts of protein extracts were immunoprecipitated with an anti-Sp1 antibody and then subjected to immunoblot with anti-NFATc1 antibody. As showed in Figure 3C the nuclear association between Sp1 and NFATc1 was significantly decreased upon CsA plus NGF treatment (-47 ± 6.0 %), suggesting that the inactivation of NFATc1 isoform, enhancing the Sp1 nuclear availability, allows the transcriptional factor to bind to the p21 promoter, leading to its transactivation (Figure 3C).

Taken together these results demonstrated that in HK-2 cells the strong cell-growth inhibition observed upon co-treatment CsA plus NGF is due to the Sp1-mediated p21 promoter transactivation. Moreover, interestingly, NFATc1 inactivation, more pronounced upon CsA+NGF treatment respect to CsA alone, exert a crucial role in increasing the Sp1 nuclear content that binding to p21 promoter enhances its activity.

### Co-treatment of CsA plus NGF triggers apoptosis via p75^NTR^

To investigate the contribution of NGF signaling pathways in CsA-induced cellular damage, we studied whether CsA plus NGF exposure modulates expression of NGF receptors.

**Figure 4:**
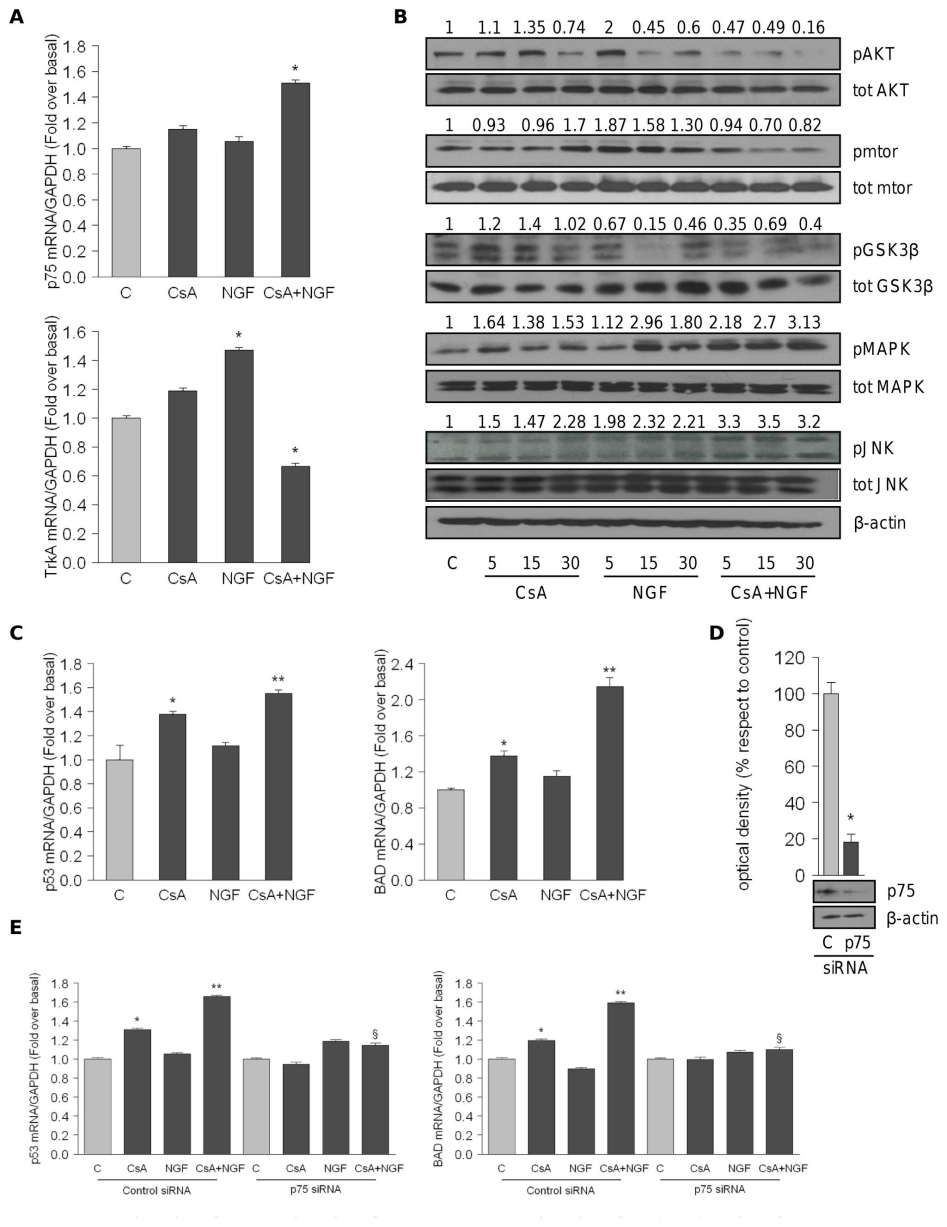
Combined treatment with CSA plus NGF activates intrinsic apoptosis via p75^NTR^. (A) Real time RT-PCR analysis of TrKA^NTR^ and p75^NTR^ mRNA treated for 24h with CsA 10nM and NGF 100ng/ml alone or in combination. *p<0.05 compared with c; **p<0.05 compared with cells treated with CsA alone. (B) HK-2 cells were untreated (c) or treated with CsA 10nM and NGF 100ng/ml alone or in combination. Levels of phosphorylated (p) Akt (Ser473) (Antibody dilution 1:1000), mtor (1:500), MAPK (Thr202/Tyr204) (1:1000), JNK (Thr183/Tyr185) (1:200) and total non-phosphorylated proteins (1:1000) were measured in cellular extracts by WB. β-Actin (1:10000) was used as loading control. Numbers over the blots represent the average fold change versus control. (C) p53 and BAD mRNA expression in HK-2 cells untreated (c) or treated for 24h with CsA 10nM and NGF 100ng/ml alone or in combination. *p<0.05 compared with c; **p<0.05 compared with CsA-treated cells. (D) WB of p75^NTR^ in HK-2 cells transfected for 72h with siRNA targeted human p75^NTR^ mRNA sequence or with a control siRNA. β-Actin was used as loading control. The upper panels represent the means ± SD of 3 separate experiments between NGF/β-Actin levels in which band intensities were evaluated in terms of optical density arbitrary units and expressed as percentages of the control (100%). *p<0.05. (E) p53 and BAD mRNA levels in HK-2 cells transfected with p75^NTR^siRNA or control-siRNA for 72 hours followed by CsA 10nM and NGF 100ng/ml treatment alone or in combination for 24 hours. *p<0.05 compared to untreated cells (c), **p<0.05 compared with CsA-treated cells; § p<0.05 compared with cells CsA+NGF-treated and transfected with control-siRNA. All mRNA sample were normalized to its GAPDH mRNA content. Bars represent the means ± SD of 3 independent experiments, each performed in triplicate. All immunoblots show a single representative of 3 separate experiments. Secondary antibodies dilutions used are: goat anti-mouse (1:2000) or anti-rabbit (1:7000) IgG or donkey anti-goat (1:3000) .

Our results, obtained by real time RT-PCR, showed that co-treatment with CsA plus NGF for 24 h induces a significant up-regulation of p75^NTR^ up to 1.5 ± 0.026 together with a down-regulation of TrkA^NTR^ receptor (0.62 ± 0.02) compared to control in HK-2 cells (Figure 4A). Concomitantly, we explored the effects of short-term stimulation with the treatments above reported on phosphorylation levels of MAPK, AKT and c-jun Kinase JNK, the main downstream effectors of the NGF signaling. As showed in the numbers on top of the blots, representing the average fold change versus control reported in Figure 4B, short exposure to NGF increased phosphorylation of AKT and its effector mTOR, MAPK and JNK. However, upon co-treatment CsA plus NGF, we observed a down-regulation of pAKT and p-mTOR and an up-regulation of pMAPK and pJNK when compared to control. Finally, the activated kinases, pGSK3β, downstream target of pAKT, and pJNK, phosphorylating cytosolic NFATc1 may contribute to inhibit the transcriptional factor and to prevent its nuclear translocation.

p75^NTR^ is currently best known for its role in mediating cell death and its death signaling cascade is mediated via one or more of the receptor’s intracellular binding partners, resulting in JNK activation and subsequent p53, BAD (Bcl-2-associated death promoter)-like proteins and caspase activation [21] Therefore, to explore the apoptotic pathway downstream of p75^NTR^, we tested, by real time RT-PCR, the expression levels of pro-apoptotic target genes, p53 and BAD, in HK-2 cells treated for 24 h with CsA and NGF alone or in combination. Our results evidenced upon combined treatment CsA plus NGF, concomitantly with an increased p75^NTR^ expression (Figure 4A), a markedly up-regulation of p53 and BAD mRNA contents (respectively 1.52 ± 0.03 and 2.0 ± 0.1, Figure 4C). In addition, in the same experimental condition, we observed a significant reduction of anti-apoptotic protein Bcl-2 mRNA levels respect to control (0.29 ± 0.026, Figure S1A). Interestingly, in the presence of p75^NTR^ knock-down (Figure 4D), our results did not showed the previous observed increasing in p53 and BAD mRNA expression levels and revealed (Figure 4E) a concomitant up-regulation of anti-apoptotic Bcl-2 mRNA (2.0 ± 0.03, Figure S1B).

**Figure 5:**
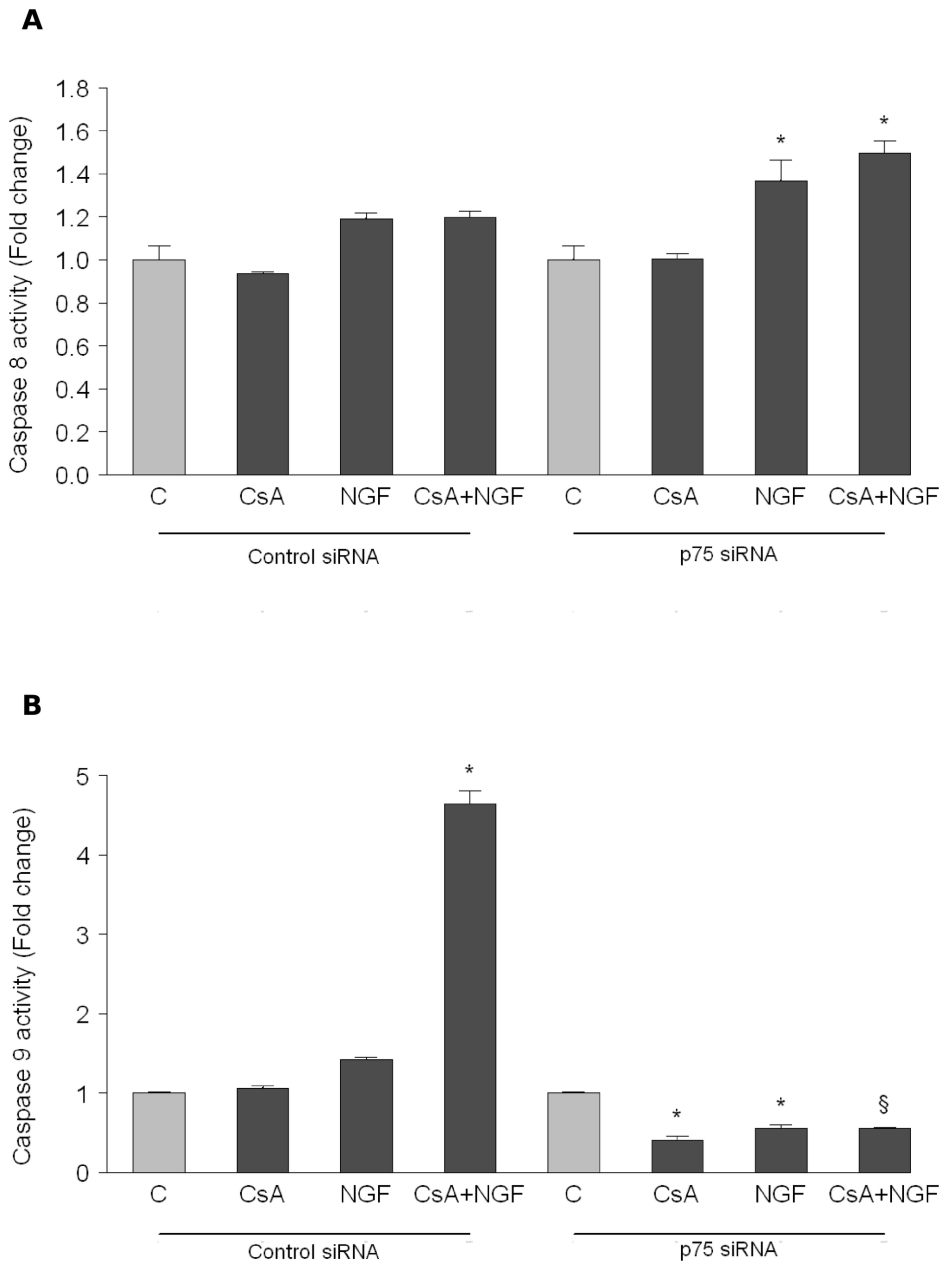
CSA plus NGF treatment activates intrinsic apoptosis via p75^NTR^. (A) caspase 8 and (B) caspase 9 activities in HK-2 cells transfected for 72 h with siRNA targeted human p75^NTR^ mRNA sequence or with a control siRNA and then treated with CsA 10nM and NGF 100ng/ml alone or in combination for 72h. *p<0.05 combined-treated versus untreated cells; § p<0.05 compared with cells transfected with a control siRNA and treated with CsA+NGF; NS: not significant. The results represent the means ± SD of 3 independent experiments, each performed in triplicate.

Finally, in order to determine which of the two apoptotic pathways were activated, we measured caspase 8 and caspase 9 activities in HK-2 cells treated with CsA and NGF alone or in combination for 72 h. As reported in Figure 5A no significant effects were elicited upon separate or combined treatment on caspase 8 activation, a marker of extrinsic apoptotic pathway. On the contrary, a strong activation of caspase 9 activity was observed when cells were treated in the presence of both CsA and NGF (4.4 ± 0.15, Figure 5B). As expected, in the presence of p75^NTR^ knock-down, caspase 9 activity was not induced (0.53 ± 0.015, Figure 5B), indicating that p75^NTR^ has a specific role in mediating the intrinsic apoptosis process enhanced by CsA plus NGF treatment in HK-2 cells.

## Discussion

The key finding of this study is that in tubular renal cells chronic exposure to NGF worsens the nephrotoxic effect exerted by CsA alone, through a dual mechanism: (i) cell growth inhibition, inducing p21 promoter activity via Sp1; (ii) promotion of intrinsic apoptosis via p75^NTR^ activation.

*In vitro* studies demonstrated that CNI-induced calcineurin-NFAT pathway inhibition, lead to increased NGF protein and gene levels, by direct NFAT involvement in NGF modulation. In accordance with previous data [19,22] our *in vitro* studies revealed that in tubular renal cells, CsA caused an increase in NGF expression (Figure 1B) via NFATc1(Figure 1C-D), suggesting that therapy with CNIs, among others, may be involved in the higher NGF serum levels detected in transplant patients [4]. 

The most intriguing and unexpected finding of our basic study is that the combined treatment with CsA plus NGF worsened the reduction in viable tubular renal cell number induced by CsA (Figure 2D). Indeed the majority of the *in vitro* and *in vivo* studies to date demonstrate that NGF exerts benefic effects in the treatment of major central neurodegenerative diseases, peripheral neuropathies, skin and corneal ulcers [1]. Moreover, NGF supply in animal models of diabetic neuropathies reverses neuropathic signs by protecting the affected PNS neurons and normalizing their activity. Therefore, initially we hypothesized that NGF might exert a protective role on tubular renal damage induced by the treatment with CsA both by promoting epithelial-mesenchymal transition via TGF-β [23] and by inducing renal cell apoptosis through five well described pathways which ultimate intersection in the activation of caspases [5]. 

Our results demonstrate that co-treatment with CsA plus NGF induced a strong up-regulation of p21 mRNA and protein levels (Figure 2D-E) concomitantly with an enhanced promoter activity (Figure 2F). Khanna et al. demonstrated that immunosuppressive drugs such as CsA induced the expression of cyclin inhibitor p21, preventing both T-cell activation and the inflammation of the graft [24]. On the other hand, some authors showed that by inducing the transcriptional activation of p21, NGF blocks PC12 cell proliferation [25,26]. Multiple transcription factor binding to sites within the human p21 promoter have been described. In particular Sp1 interacts with specific regulatory elements and plays a key role in cell cycle arrest and apoptosis in carcinoma cells [27-29]. Of interest, we observed that in our experimental conditions, the transactivation of the p21 promoter was abrogated in presence of mithramycin (Figure 2F), highlighting that Sp1 is important for the up-regulatory effect induced by the co-treatment on p21 expression in HK-2 cells. In line with that reported by Wang et al, demonstrating that in human intestinal cells the activated NFATc1 acts as a negative regulator of Sp1 nuclear translocation [30], we identified with the inactivated NFATc1 the common target through which the combined treatment increased Sp1 binding on its responsive elements located in p21 promoter, enhanced promoter transcriptional activity. Interestingly, in our experimental cellular model, NFATc1 nuclear translocation was inhibited both through CsA-induced calcineurin inhibition and by NGF-signaling (Figure 3A-B) . Indeed in HK-2 cells the modulation of TrkA^NTR^ and p75^NTR^ expression levels induced by CsA plus NGF exposure, led to the activation of two kinases, GSK3β and JNK (Figure 4B) that, phosphorylating cytosolic NFATc1, caused an arrest of its nuclear translocation. As revealed by immunoprecipitation assays (Figure 3C) the co-treatment, reducing the physical interaction between NFATc1 and Sp1 in nuclear compartment, enhanced the nuclear content of Sp1 able to binding to its responsive elements containing in p21 promoter inducing its transcriptional activity. 

All together these results demonstrate that the simultaneous strong inactivation induced by CsA and NGF on NFATc1 nuclear translocation allows the binding of Sp1 on p21 gene promoter whose transactivation arrests HK-2 cells growth. Thus, these data suggest that this may represent a new described molecular mechanism by which NGF worsens the tubular renal damage induced by CsA.

Secondly, the strong up-regulation of p75^NTR^ observed in cells treated with CsA plus NGF (Figure 4A), prompted us to investigate whether activated p75^NTR^-signalling promoted tubular renal cell apoptosis, contributing to worsening CsA-induced tubular cell damage. In neural cells p75^NTR^ plays an ambiguous “Jekyll-and-Hyde” role, being able to either kill or stimulate cell survival and differentiation [31,32]. Some authors speculated that the increased p75^NTR^ expression observed in patients with multiple sclerosis [33], Amyotrophic Lateral Sclerosis [34] and Alzheimer’s disease [35] may be involved in these neurodegenerative diseases. It is important to note that the pro-apoptotic actions of p75^NTR^ may not be restricted to the neural system, as *in vitro* and *in vivo* studies indicate a role for p75^NTR^ in apoptosis within non-neuronal cells [36-39]. Although the mechanism by which p75^NTR^ -mediated apoptosis is activated has not been fully elucidated, many studies have shown that the stress activated MAP kinase and the activation of JNK signaling, a downstream consequence correlated with p75^NTR^ activity, triggers apoptosis [40]. Interestingly, our results evidenced that the increased JNK phosphorylation levels resulting from p75^NTR^ up-regulation obtained upon co-treatment, leads to a strong increase of target apoptotic genes as p53 and BAD (Figure 4C-D) and to a reduced expression of the anti-apoptotic gene Bcl-2 (Figure S1A). Moreover, according to the literature, indicating that p75^NTR^ -induced death appears to involve the activation of caspases 9 and 3 but not caspase 8 [41], we verified that in our CsA plus NGF-treated culture model, the intracellular death-pathway induced by p75^NTR^ activation is mediated by intrinsic apoptosis, as we observed an increase of caspase 9 activity (Figure 5B) whereas caspase 8 was not induced (Figure 5A). 

Finally, our findings were supported by small inhibitory RNAs (siRNA) directed silencing of p75^NTR^ (Figure 4D), we found that the increased p53 and BAD mRNA expression levels were reversed (Figure 4E), together with the abrogation of the intrinsic apoptotic signal (Figure 5B), highlighting the contribution of NGF/ p75^NTR^ axis in mediating HK-2 apoptosis. These latter results suggest that NGF-p75^NTR^ -activated signaling represents an additional new mechanism through which CsA, upon NGF exposure, exerts its nephrotoxic effect in tubular renal cells. 

In summary, our *in vitro* model provides new data on NGF as an inducer of growth arrest and apoptosis in tubular renal cells exposed to CsA. Further *in vivo* studies are necessary to establish whether NGF and its receptors are involved in the progression of graft damage resulting from CNI-induced nephrotoxicity. It still remains to be elucidated whether in patients treated with CNI because of autoimmune or chronic inflammatory diseases as well as in kidney transplantation, the detection of high NGF serum levels could be considered a negative prognostic marker. 

## Supporting Information

Figure S1 **Co-teatment CsA plus NGF activates antiapoptotic gene Bcl-2 via p75^NTR^.** (A) BcL2 mRNA expression in HK-2 cells untreated (c) or treated for 24 h with CsA 10nM and NGF 100ng/ml alone or in combination. Each sample was normalized to its GAPDH mRNA content. *p<0.05 compared with c; **p<0.05 compared with cells treated with CsA alone. The results represent the means ± SD of 3 independent experiments, each performed in triplicate. (B) BCL-2 mRNA levels in HK-2 cells transfected with p75^NTR^ siRNA or control siRNA for 72 hours followed by CsA 10nM and NGF 100ng/ml treatment alone or in combination for 24 hours. Each sample was normalized to its GAPDH mRNA content. *p<0.05 compared to c, **p<0.05 compared with cells treated with CsA alone; § p<0.05 compared with cells treated with combined treatment CsA plus NGF and transfected with control siRNA. The results represent the means ± SD of 3 independent experiments, each performed in triplicate.
(TIF)
