# Supplementary figures and images for: Exposure to Nerve Growth Factor Worsens Nephrotoxic Effect Induced by Cyclosporine A in HK-2 Cells
Source: PLoS One. 2013 Nov 7;8(11):e80113. doi: 10.1371/journal.pone.0080113 (PMC3820545; doi:10.1371/journal.pone.0080113)

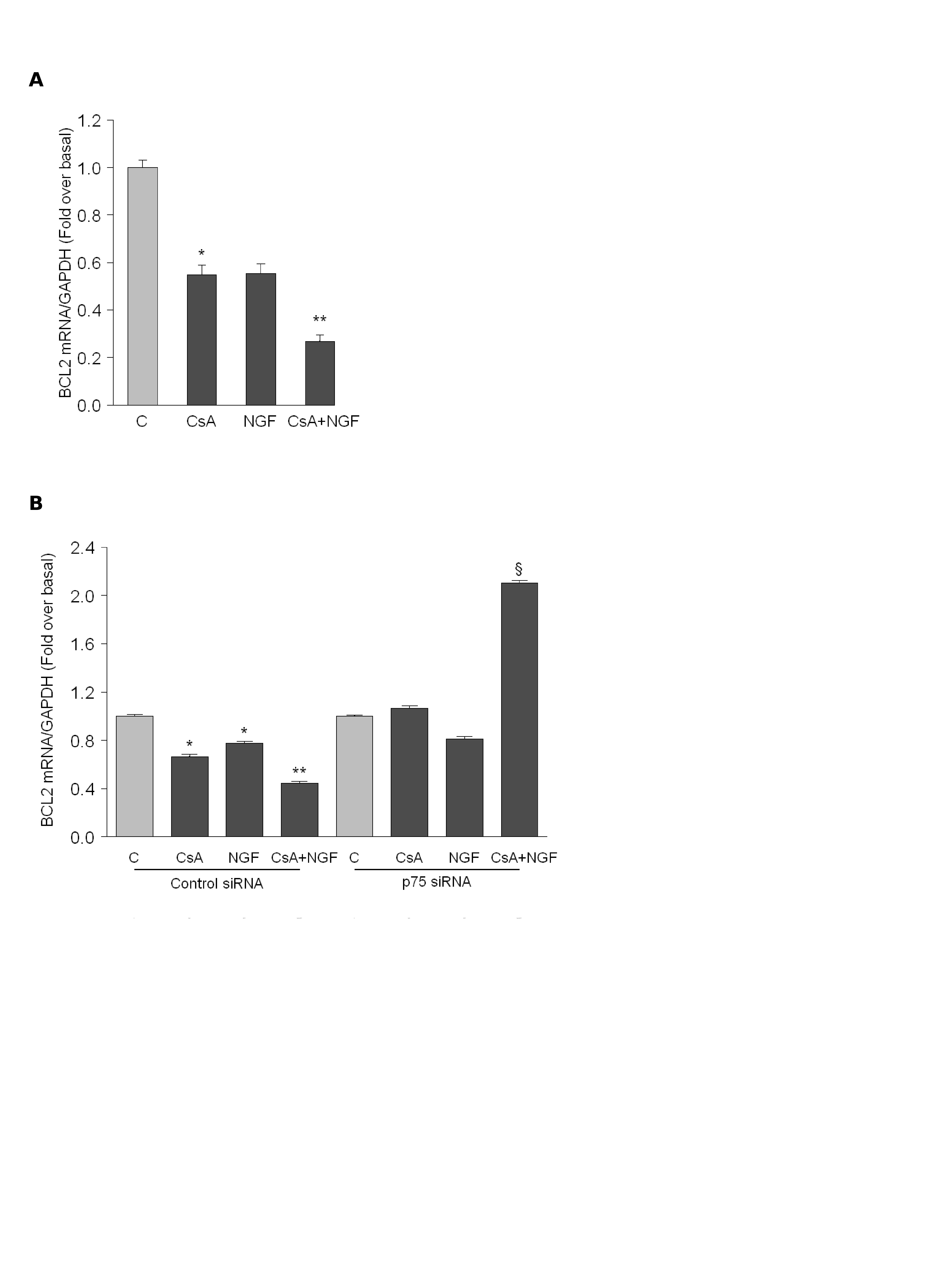

Supplement: Figure S1 — Co-teatment CsA plus NGF activates antiapoptotic gene Bcl-2 via p75NTR. (A) BcL2 mRNA expression in HK-2 cells untreated (c) or treated for 24 h with CsA 10nM and NGF 100ng/ml alone or in combination. Each sample was normalized to its GAPDH mRNA content. *p<0.05 compared with c; **p<0.05 compared with cells treated with CsA alone. The results represent the means ± SD of 3 independent experiments, each performed in triplicate. (B) BCL-2 mRNA levels in HK-2 cells transfected with p75NTR siRNA or control siRNA for 72 hours followed by CsA 10nM and NGF 100ng/ml treatment alone or in combination for 24 hours. Each sample was normalized to its GAPDH mRNA content. *p<0.05 compared to c, **p<0.05 compared with cells treated with CsA alone; § p<0.05 compared with cells treated with combined treatment CsA plus NGF and transfected with control siRNA. The results represent the means ± SD of 3 independent experiments, each performed in triplicate. (TIF) [file pone.0080113.s001.tif]
